# A new set of 182 microsatellites for *Eucalyptus*: characterization and mapping in a four-species consensus linkage map

**DOI:** 10.1186/1753-6561-5-S7-P34

**Published:** 2011-09-13

**Authors:** Danielle Faria, Eva Mamani, Juliana Sena, Alexandre Alves, Clarissa Falcao, Rodrigo Lourenço, Georgios Pappas, Dario Grattapaglia

**Affiliations:** 1 Plant Genetics Laboratory, EMBRAPA Genetic Resources and Biotechnology, PqEB, CEP 70770-970, DF, Brazilia, Brazil; 2 Plant Genetics Laboratory, EMBRAPA Genetic Resources and Biotechnology, CEP 70770-970, DF, Brazilia, Brazil, and Graduate Program in Genomic Sciences Biotechnology and Universidade Católica de Brasília, SGAN Qd 916, CEP 70790-160, DF, Brasília, Brazil

## Background

*Eucalyptus* is the most widely planted hardwood crop in the tropical and subtropical world. Plantations of Eucalyptus species supply high-quality wood for industrial applications and are important sources of carbon neutral renewable energy in Brazil. *E. grandis* and *E. urophylla* and their hybrids are the most widely planted species in fast growing commercial forests in Brazil. *E. globulus* is the preferred raw material by the mills generating a pulp that is considered superior by the market. However as a pure species it does not grow adequately in Brazil but performs well in hybrid combinations. Breeding programs have increasingly incorporated *E. globulus* germplasm in fast moving elite populations. Molecular breeding in such populations will require information on markers, comparative mapping and QTL validation across pedigrees involving these different species. Highly multiallelic and transferable microsatellites not only are excellent tools for individual identification, but also provide robust and efficient framework genetic maps that serve well for mapping thousands of bialellic higher throughput markers such as Single Nucleotide Polymorphisms (SNPs) and Diversity Array Technology (DArT). Furthermore microsatellites provide a powerful way for QTL validation across species. We describe the development and characterization of 182 new microsatellites, most of them derived from ESTs and some from a genomic shotgun library. These markers, together with other previously developed ones were used to build a consensus map involving three different pedigrees derived from intercrossing four species of *Eucalyptus*.

## Methods

The source of ESTs used to mine microsatellites and the methods used to select loci and design primers were described earlier [1,2]. Data from a sample sequencing experiment, carried out back in 2003 using a Sanger sequenced shotgun genomic library, was used for microsatellite discovery (R.T. Lourenço, unpublished). Microsatellite, primer pairs were initially screened for assay success, polymorphism and inter-specific transferability by colorimetric detection on polyacrylamide gels. Selected loci had their forward primer re-synthesized with a fluorescence label and used in downstream genotyping. Loci were characterized using a set of 32 unrelated individuals each of *E. grandis* and *E. globulus*. Map construction was carried out for three different pedigrees of *Eucalyptus* one of them being the pedigree used earlier for a reference map construction [3]. Mapping was conducted using JoinMap 3.0 [4]with a minimum LOD score of 10. and marker order determined with the default mapping module parameters. Parameters of genetic information content (PIC polymorphism information content; PE = probability of paternity exclusion and PI = probability of identity) were estimated for all newly developed microsatellite markers for each species separately.

## Results

Data mining of 22,298 unigenes provided 1,261 microsatellite loci. Most of them were (54.71%) trinucleotide repeats. After all screening steps, 494 microsatellites amplified discrete and polymorphic fragments and 210 were selected for fluorescence based genotyping. From the single-pass genomic shotgun library 41 microsatellites were also selected following then same screening steps. Genetic maps were initially built for each pedigree separately. A consensus map was then built by combining separately the homologous linkage groups of the three maps. The total number of microsatellite markers eventually mapped was 448 including the 234 previously mapped [3]and part of those published but not yet mapped earlier [1,2]. The map had an estimated total recombination distance of 1,297 cM and an average distance between adjacent markers of 3.48 cM. The linear order of markers was conserved on most linkage groups across the individual pedigree maps and the consensus map.with no evidence of rearrangement of chromosomal blocks. For the 182 newly developed microsatellites characterized the allele size range did not vary between *E. grandis* and *E. globulus*. Only 15 loci for *E. grandis* and 17 loci for *E. globulus* were monomorphic in the set of individuals genotyped. The average number of alleles was very close in the two species, 4.6 for *E. grandis* and 4.8 for *E. globulus*. A significant estimate of null allele frequency was found for only seven loci and these coincided for the two species Average PIC, PE and PI for the set of loci were not substantially different between the two species. Furthermore the overall performance of the EST derived and genomic shotgun derived sets of microsatellite did not show any appreciable difference, somehow unexpectedly due to the generally lower polymorphism of genic microsatellites. The ascertainment bias introduced by the much larger set of EST derived microsatellites and rigorous screening applied might have contributed to this.

## Conclusion

This work summarizes the development and characterization of a new set of 182 new microsatellites markers and presents a relatively dense microsatellite marker consensus map involving four different species of *Eucalyptus*. The new 182 microsatellites developed are robust and polymorphic enough to be used for applications in breeding programs that involve individual identification as well as for comparative QTL mapping and marker assisted selection.
